# The oblique effect in visual working memory is enhanced by distraction, regardless of tDCS manipulations

**DOI:** 10.3758/s13415-026-01443-z

**Published:** 2026-04-21

**Authors:** Harun Yörük, Benjamin J. Tamber-Rosenau

**Affiliations:** 1 Department of Psychology, University of Houston, Houston, TX USA; 2 Department of Psychological Sciences, Purdue University, 703 3rd Street, West Lafayette, IN 47907 USA

**Keywords:** Visual working memory, Transcranial direct current stimulation, Oblique effect, Visual distraction, Sensory recruitment, Frontoparietal

## Abstract

The sensory recruitment model suggests that visual working memory (VWM) relies on early visual cortex (EVC), while the alternative frontoparietal account argues VWM representation is independent from EVC. An emerging consensus suggests that VWM relies on a distributed network, with different brain loci serving behavior depending on task demands. To evaluate VWM reliance on EVC vs. parietal cortex, we applied anodal (or sham) transcranial direct current stimulation (tDCS) to either occipital cortex (Oz) or right posterior parietal cortex (P4) to manipulate cortical representations before an orientation VWM task. To provoke perceptual interference and encourage reliance on parietal cortex, in half of blocks, distractors appeared during VWM delays. We predicted that parietal tDCS would improve VWM capacity to protect representations against ongoing distractions. We also expected that occipital tDCS could either increase memory capacity in nondistraction blocks or increase susceptibility to ongoing distraction. Finally, we reasoned that the oblique effect, which is thought to rely in part on EVC, would be reduced by distractor presence and the accompanying shift to reliance on parietal representations. Contrary to our predictions, neither tDCS manipulation affected VWM reports and the oblique effect was always present but was paradoxically stronger with distraction. These results could suggest reliance on EVC for VWM maintenance, even under distraction. Alternatively, these results could signal loss of fine-grained VWM detail under distraction due to use of representational strategies that maximize task performance rather than veridical orientation representation.

**Supplementary Information:**

The online version contains supplementary material available at 10.3758/s13415-026-01443-z.

Visual working memory (VWM) is the system that functions for short-term storage and manipulation of visual information after the perceptual input is no longer available. Current debates contrast two main accounts of VWM’s neural underpinnings and general mechanisms. The *sensory recruitment model* suggests that VWM representations rely on the sensory code in early visual areas (occipital cortex) (Adam et al., 2022; Harrison & Tong, 2009; Serences et al., 2009). The *frontoparietal account* suggests that VWM representations are mostly maintained in frontoparietal areas (Bettencourt & Xu, 2016; Roth et al., 2006; Srimal & Curtis, 2008; Todd et al., 2011), with occipital representations being epiphenomenal, or at least having little influence over behavior. An additional account, the *distributed networks view* (Adam et al., 2022; Christophel et al., 2017)*,* offers a potential reconciliation, suggesting VWM representations can be transferred across occipital and frontoparietal regions based on task demands (Xu, 2025). The present research aimed at a new test of these accounts by adapting a task from the fMRI literature, which is inherently correlational, to a noninvasive neural stimulation approach that can test causal hypotheses.

## Neural accounts of visual working memory

The *sensory recruitment model* suggests that maintenance of VWM representations depends on representations in the early visual cortex, the same neural regions that initially encode incoming perceptual stimuli (Harrison & Tong, 2009; Serences et al., 2009). Sensory recruitment can explain the efficient processing of VWM representations by suggesting that both perceptual and VWM information are represented with high fidelity in the visual cortex. Therefore, the system does not need separate copies for different stages of visual information processing and instead can represent both sensory and mnemonic information efficiently using neural circuits that are specialized for visual information (Adam et al., 2022). Multivariate analysis of functional magnetic resonance imaging (fMRI) studies showed that the contents of VWM can be decoded from activity in the visual cortex when the perceptual stimuli are no longer available. Specifically, Harrison & Tong (2009) decoded the orientation of a peripherally-presented visual item stored in VWM during a delay period throughout contralateral visual areas V1-hV4, showing representation in the brain regions that also represent the item-present visual field in the early visual cortex. Ester et al. (2009) observed decoding of VWM contents from both the contralateral and ipsilateral visual cortex to the stimulus location, showing that VWM representations can be flexibly represented without spatial specificity, but still in the early visual cortex. However, when the VWM task required location-bound representations, classification of memory activity from contralateral visual areas was higher than the ipsilateral areas to the stimuli, suggesting spatially specific encoding of VWM representations when dictated by task demands (Pratte & Tong, 2014). Thus, the strict version of the sensory recruitment model would require both perceptual and VWM representations to be maintained in the same retinotopic neural population. However, current findings point towards a more flexible form of the sensory recruitment model where VWM representations are represented globally, still in the early visual cortex but not necessarily in the identical neural populations (Ester et al., 2009; Rademaker et al., 2019) unless task requirements lead to more spatially focal representations (Yörük et al., 2020).

Contrary to the sensory recruitment model, the *frontoparietal account* favors the idea that the VWM representations that guide behavior are localized to frontoparietal cortex and that other brain regions that show signals correlated with VWM play nonrepresentational roles. There is ample evidence showing that frontoparietal areas maintain and store VWM representations, without requiring visual cortex activity (Ester et al., 2015; Roth et al., 2006; Srimal & Curtis, 2008; Todd et al., 2011; Xu & Chun, 2006). Neural evidence via fMRI showed that frontoparietal network activity correlates with VWM maintenance (Roth et al., 2006), and temporal activation of the lateral prefrontal cortex correlates with VWM encoding (Todd et al., 2011). Visual working memory representations can be decoded from the inferior intraparietal sulcus (IPS) during VWM encoding and maintenance (Bettencourt & Xu, 2016), and activity in the posterior parietal cortex (PPC) reflects the storage capacity of VWM (Todd et al., 2011). Also, the decoding of VWM representations extends to frontal cortical areas, with evidence of the frontoparietal network encoding the goal-oriented representations of VWM items but with coarser detail compared with more posterior cortex (Ester et al., 2015). The most important critique of the sensory recruitment model addressed by the frontoparietal account is that if the same cortical areas are processing perceptual and memory information at the same time, ongoing perception should disrupt the maintenance of VWM representations (Xu, 2017). Bettencourt & Xu (2016) tested this claim (which we revisit below in explaining the present study’s approach) with an fMRI study showing that VWM representations in a grating orientation VWM task can be decoded via multivariate pattern analysis (MVPA) in the superior intraparietal sulcus (IPS) regardless of whether there was a distractor present or not. However, decoding of VWM representations in the occipital cortex was significantly reduced by distractor presence. This distraction effect was not reflected in behavior, only in the occipital decoding. They concluded that IPS (in the posterior parietal cortex [PPC]) is the main storage area for VWM, with occipital activity not guiding behavior, thus arguing against the sensory recruitment account and in favor of the frontoparietal account. A further study by Rademaker & colleagues (2019) showed that visual distraction can indeed interfere with both behavioral performance and the sensory neural code for VWM representations in early visual cortex. However, this interference was dependent on the distractor type. Contrast-reversing noise distractors did not interfere with neural code or the behavioral performance, but flickering picture (face and gazebo) distractors did interfere with both behavior and the neural code. Rademaker & colleagues (2019) concluded that early visual cortex contributes to processing of VWM representations, especially when high-fidelity representations needed to be sustained. Nevertheless, Xu (2020) has until recently argued that any signal observed in the early visual cortex is epiphenomenal and not essential for VWM storage. Instead, Xu (2020) has suggested that it might be an extension of encoding, or serve a different role than storage, such as a template of VWM representation to be utilized for target-to-probe comparisons, although a recent work also suggests that transformed sensory information could be stored in early visual cortex in accordance with task demands (Xu, 2025), similar to the more flexible form of sensory recruitment proposed by Yörük & colleagues (2020).

The accounts discussed above propose that either the sensory cortex is the main storage area for VWM or parietal (and perhaps frontal) cortex is the essential area for VWM storage. However, there is evidence that the contents of VWM are represented across a wide cortical network spanning visual, parietal, temporal, and frontal cortices (Christophel et al., 2017), calling into question the notion of a single locus for VWM storage. These observations are consistent with the *distributed networks view*, which suggests that the neural basis of VWM depends on multiple factors, such as format of the VWM representation and the specific VWM function (e.g., storage, attentional selection, manipulation, protection against interference), with the resources that support these different formats and functions distributed across multiple brain regions (Lorenc & Sreenivasan, 2021). Which of these resources are used to maintain VWM might depend on the task and VWM representation format. For example, VWM representations might be sustained with a “sensory code” in the early visual areas when low-level sensory details are needed for the task at hand. Indeed, our past research has shown that VWM representations rely on a sensory code that is influenced by the retinotopic structure and organization of early visual cortex when low-level sensory details must be bound to one another—e.g., orientation and location (Yörük et al., 2020). However, sensory influence was diminished when spatially specific encoding of the stimuli was no longer required for task performance (Yörük et al., 2020; Harrison & Bays, 2018), suggesting flexible use of sensory codes or more abstract codes as demanded by the task. Conversely, higher level cortices might be used for categorical or abstract VWM representation formats (Christophel et al., 2017). The distributed networks view also brings the idea of “parallel coding” for VWM. Previous research found that neural coding for the VWM representations can be found in both visual and frontoparietal cortices, in parallel (Lorenc et al., 2018; Riley & Constantinidis, 2016). Serences (2016) suggested that these parallel codes can be used flexibly based on the task demands, such as in anticipation of distraction (Lorenc et al., 2018). Even though having two separate copies for the same VWM representation might seem redundant, the parallel code in the parietal areas can provide protection against incoming visual interference (Bettencourt & Xu, 2016; Linden et al., 2012), suggesting an important functional role for this redundancy.

In summary, underlying neural mechanisms for VWM representations can be found both in the early visual cortex and frontoparietal areas in the brain. The main resource for VWM storage might be the early visual cortex when spatially specific representations or other fine details are needed, but the parietal cortex could supersede early visual cortex in guiding behavior when there is a need to protect memoranda from ongoing perception or when more abstract representations may suffice to guide behavior. Considering across lines of research, it appears that VWM representations can be represented flexibly across multiple cortical regions depending on the task demands, which is most consistent with the distributed networks view.

In previous studies, we evaluated evidence for the sensory recruitment account by using visual (perceptual) crowding and a parallel phenomenon in visual working memory to make inferences about neural basis of VWM representations via behavioral measures. Visual perceptual crowding is an impairment in the identification of a stimulus in peripheral vision when it is surrounded by flankers, and it depends on the organization and structure of the early visual cortex (Whitney & Levi, 2011). We found that crowding also occurs in VWM, even when stimuli are presented in a sequence, and thus spatial interactions can occur only in memory. The finding that the specific crowding phenomenon of radial-tangential anisotropy can happen during VWM maintenance (not encoding) thus demonstrates that VWM maintenance is influenced by the retinotopy of the early visual cortex (Harrison & Bays, 2018; Yörük et al., 2020; Yörük & Tamber-Rosenau, 2022). In the present study, we similarly use a behavioral hallmark of representation in early visual cortex to evaluate the representational basis of VWM during different task and brain stimulation conditions. Specifically, we used the magnitude of the oblique effect—the higher selectivity and better identification for cardinal (horizontal and vertical) orientations than oblique orientations—to determine whether early visual cortex representations are guiding behavior, because the oblique effect is known to arise from early visual cortex (Berkley et al., 1975; Furmanski & Engel, 2000; Li et al., 2003). In addition, past research also showed that the magnitude of the oblique effect can reflect strategy or ability differences between individuals with respect to what neural resources they use for VWM maintenance. Specifically, Keogh & colleagues found that VWM performance of aphantasic individuals (Keogh & Pearson, 2018)—who do not report experiencing visual imagery—showed no oblique effect, and these individuals reported nonvisual strategies to maintain VWM representations even though their VWM capacities did not differ from the non-aphantasic individuals (Keogh et al., 2021). Because visual imagery is supported by representations in visual cortex (Pearson, 2019; Sunday et al., 2018), these findings suggested distinct strategies can be used to maintain VWM. Therefore, we aim to leverage the oblique effect as a phenomenon that can be used to test visual cortex-dependent vs. parietal-dependent strategies for VWM representation.

### Transcranial direct current stimulation and visual working memory

One goal of the present research is to test the accounts of VWM described in the preceding section by providing causal evidence for the neural basis of VWM representations. Even though there is ample evidence supporting both the sensory and frontoparietal accounts of VWM (and consistent with the distributed networks view as well), most studies on the neural basis of VWM representation have used indirect or correlational methods. Moreover, causal neural evidence for the sensory recruitment model has thus far proved elusive. For example, a recent meta-analysis of transcranial magnetic stimulation (TMS) studies on sensory areas in visual working memory concluded that there was evidence for the sensory recruitment model (Phylactou et al., 2022), but this meta-analysis relied on unsigned effect sizes (such that effects in opposite directions summed instead of canceling out), calling into question this conclusion. Phylactou et al. (2022) also computed a meta-analysis of signed effect sizes and in that analysis did not find overall evidence of VWM performance modulation by TMS.

Another method that can give insight about how neural modulation can affect behavior and cognitive processing is transcranial direct current stimulation (tDCS). Transcranial direct current stimulation is a noninvasive method to manipulate the excitability of the neurons in a particular brain region by applying a weak direct current (Reinhart et al., 2017; Thair et al., 2017). The effects of tDCS persist after stimulation. The specific duration of the prolonged tDCS effects might depend on various factors, such as stimulation duration, intensity, and targeted brain location, and can last up to 5 hr (Reinhart et al., 2017). For example, a single-session 20-min stimulation on the visual cortex was found to have effects lasting 1.5 hr (Reinhart et al., 2016). Transcranial direct current stimulation uses an anode (consistently positive terminal) and a cathode (consistently negative terminal) to apply up to a typical limit of 2 mA of direct current. In most circumstances, brain regions under the anode electrode are expected to have enhanced activity, while brain regions under the cathode are expected to have reduced activity (Nitsche et al., 2008).

Even though there are studies in the literature that investigated the effects of tDCS on VWM performance, whether tDCS has an effect and the direction of any such effect are not clear. Anodal stimulation of posterior parietal cortex (PPC) improved VWM capacity (Li et al., 2017; Tseng et al., 2012) but not the control of attention against distractors (Li et al., 2017). Some studies reported individual differences or greater effects when capacity was overtaxed: low performers benefitted from anodal stimulation the most on the right PPC (Hsu et al., 2016), and tDCS on right PPC increased performance only when the item capacity was exceeded with a set size of 6 (Wang et al., 2019). There is also evidence showing that cathodal tDCS on parietal cortex reduces the capacity of VWM (Heimrath et al., 2012). However, another study showed that the precision of VWM was enhanced both by anodal and cathodal stimulation of the right PPC (Heinen et al., 2016). Therefore, whether VWM benefits from anodal or cathodal stimulation is the subject of mixed findings. Other studies failed to replicate beneficial tDCS effects on VWM performance: Robison & colleagues (2017) failed to replicate the findings about selective role of right PPC from Li & colleagues (2017). Jiang & colleagues (2024) failed to replicate the findings from Wang et al. (2019) despite using a larger sample size, a larger number of trials, and complete counterbalancing of tDCS sessions. Findings about the effect of tDCS on the occipital cortex for VWM performance are even more limited than those on PPC. One study showed that anodal tDCS to the occipital cortex improved VWM performance only when the encoding duration was short, suggesting that the early visual cortex was essential for VWM encoding and consolidation but not retention (Makovski & Lavidor, 2014). Thus, while tDCS holds promise for investigation of the neural basis of VWM, the promise remains unfulfilled as of yet.

### The present study

The present study aimed to address the premise that VWM representations can be represented flexibly across the cortical network depending on the task demands, consistent with the distributed networks view reviewed above. We thus aimed to test both sensory and parietal accounts of VWM under varying stimuli and task conditions that we expected to manipulate the balance of dependence on either parietal or occipital representations to report the contents of VWM. Importantly, the design of this research could allow support for any of the three accounts reviewed: sensory recruitment, frontoparietal, or distributed networks.

As described above, Bettencourt & Xu (2016) showed evidence for orientation VWM representations in superior intraparietal sulcus (IPS) regardless of whether there were (gazebo image) distractors presented during the delay period. This contrasted with occipital representations, which appeared to be abolished by the distractors without any impact on behavior. A further study (Rademaker et al., 2019) also found similar reduction in occipital representations with meaningful pictures as distractors. Critically, Rademaker & colleagues (2019) observed accompanying behavioral decrements, suggesting a more important role for occipital cortex in supporting VWM. These conflicting results using imaging drove the present study’s aim to causally manipulate either PPC (Experiment 1) or occipital cortex (Experiment 2) during an orientation VWM task, either with or without delay-period distraction.

## Experiment 1

In Experiment 1, we aimed to investigate the role of PPC in representing memoranda during distraction and/or protecting memoranda against incoming perceptual input. Specifically, we tested if enhancing activity in PPC via anodal tDCS would increase overall VWM performance, especially during incoming sensory input, when VWM should most rely on PPC because of the presence of distraction (Bettencourt & Xu, 2016). We also aimed to understand whether that enhancement might lead participants to rely on VWM representations maintained in PPC instead of early visual cortex, as discussed in the general introduction (above).

We expected an overall enhancement of VWM performance with anodal stimulation of PPC considering the previous tDCS findings in VWM. Based on the frontoparietal account of VWM, we additionally expected enhancement from tDCS to reduce the oblique effect, because we hypothesized that tDCS would potentiate PPC and lead participants to rely on PPC for VWM. Reliance on PPC would effectively push all participants to rely on the highly successful, oblique-effect-free strategy described in aphantasics by Keogh & colleagues (2021), regardless of their imagery abilities.

Following the distributed network account of VWM, we also expected a main effect of distractor presence. Specifically, we expected the oblique effect to be weaker in trials with visual distraction during the memory delay, when the system relies on parietal areas instead of early visual cortex to maintain VWM representations due to the incoming visual stimuli (Bettencourt & Xu, 2016). Conversely, in distractor absent conditions, the oblique effect should be observed because the parietal cortex is not needed to protect the representations from incoming visual input, and behavior could thus rely on early visual cortex representations, which should be more susceptible to the oblique effect (Berkley et al., 1975; Furmanski & Engel, 2000; Li et al., 2003).

Conversely, the sensory recruitment account of VWM would predict that we should not observe any enhancement from the tDCS on PPC. Under this account, distractor presence should hinder VWM performance overall, and the oblique effect should be observed regardless of the distractor presence.

### Participants

Thirty-seven participants from the University of Houston community (6 males, 31 females, mean age 20.37) were recruited. The initial cohort consisted of 24 participants. After the main effect of the tDCS condition was analyzed, a Bayesian stopping rule with maximal sample size (Schönbrodt & Wagenmakers, 2018) was applied to reach Bayes factors that conservatively allow clear interpretations (BF > 5 for presence of effects or BF < 1/5 for absence of effects; contrast with the more typical approach of BF > 3 or BF < 1/3). Additional participant groups of four were recruited, and data collection was planned to be stopped when the sample size was increased by 50% regardless of achieved BF. It should be noted that this optional-stopping procedure does not inflate Type I error as a similar procedure would in a frequentist statistical analysis (Rouder, 2014). The main effect of tDCS remained inconclusive (BF = 0.839) after reaching the planned 50% maximum increased sample size that governed our stopping rule. The study was conducted under a protocol approved by the University of Houston Institutional Review Board. The participants were compensated via either extra credit or gift cards for Amazon.com. Two participants were excluded from the analyses for having chance-level performances in at least one of the experiment sessions (accuracy ≤ 50% in overall); therefore, the following analyses were conducted with 35 participants (6 males, 29 females, mean age 20.37).

### Procedure

At the beginning of each session, participants received either anodal or sham tDCS (manipulated across sessions within subjects). The tDCS was administered using a transcranial electric stimulator machine (Soterix Medical 1x1 transcranial Electrical Stimulation, 1x1-tES Model: 2001), powered by two 9V batteries. Conductive rubber electrodes were placed in a conductive wet sponge (5 cm x 7 cm) that was soaked with 6 milliliters of 0.9% saline (NaCl) solution on each side, and the sponge was placed on the surface of the skin under headbands. The anodal electrode was placed on the right PPC (P4) region based on the 10/20 EEG system, and the cathodal reference electrode was placed on the left cheek, for 20 min with 2.0 mA intensity (or 0 mA during sham, excepting ramp up/ramp down stimulation at the beginning and end to disguise sham stimulation). The cheek is suggested to be a safer option for the reference electrode placement, due to its relative proximity to the brain, and because it is not directly involved in cognition (Berryhill et al., 2010; Reinhart et al., 2017; Tseng et al., 2012). Also at least 8 cm distance between electrodes has been suggested to avoid shunting effects (Thair et al., 2017; Wagner et al., 2007) and to avoid the current travel through the cerebrospinal fluid without stimulating the cortex (Moliadze et al., 2010); this criterion is also satisfied by the use of the cheek for the cathodal electrode. The order of anodal and sham sessions was counterbalanced across participants.

**Fig. 1:**
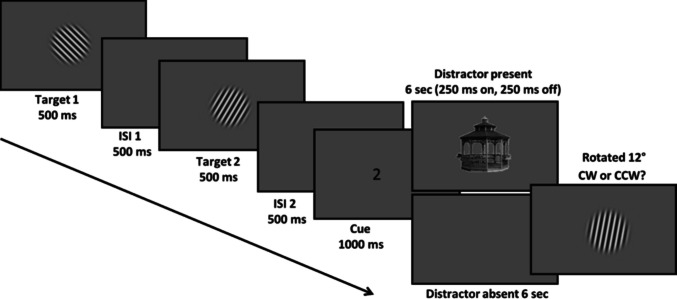
Trial sequence of the retro-cue orientation report task in Experiments 1 and 2

Immediately following the tDCS stimulation and removal of electrodes, participants completed a VWM task adapted from the tasks used by Bettencourt & Xu (2016) and Harrison & Tong (2009) to be able to link the causal inferences potentially supported by tDCS to prior, noncausal results from other techniques. In each trial of this study, participants saw two oriented Gabor patches (80% contrast) sequentially presented at the center of the screen. The background of the screen was gray. Each Gabor patch was presented for 500 ms with a 500-ms interstimulus interval. Patch orientations were chosen at random except that if the first orientation was left-tilted (tilted counterclockwise from vertical), the second orientation was right tilted (tilted clockwise from vertical), and vice versa. Randomly determined orientations were binned into cardinal and oblique categories based on whether the target orientation was closer to cardinal angles (near vertical: between 0–22.5° and 157.5°–180°, or near horizontal: 67.5°–112.5°) or oblique angles (22.5°–67.5° and 112.5°–157.5°). After another 500-ms interval, participants saw a 100% valid retro-cue (the digit 1 or 2, presented at central fixation) indicating whether they needed to remember the first or second Gabor. During the 6 s of VWM delay, participants either saw on and off flashing (250-ms phases) gazebo pictures as ongoing perceptual distraction, or they were presented with a blank screen. Distractors consisted of 12 different gazebo pictures borrowed from the VWM task used in the Rademaker & colleagues’ study (Rademaker et al., 2019), cycling randomly. At the response phase, a Gabor patch was presented that was rotated 12 degrees^1^ clockwise or counterclockwise compared to the cued item, and the participants reported the rotation direction. Participants were instructed to press the right arrow key on the keyboard if the response probe was rotated clockwise compared to the target stimulus, and the left arrow key if it was rotated counterclockwise. There were 256 trials in each session, consisting of interleaved 32-trial distractor-present and 32-trial distractor-absent blocks. There were two sessions in total (anodal tDCS, sham). Before the application of tDCS participants started by completing an 8-trial (4 distractor-present and 4 distractor-absent) training block to get familiar with the task. Familiarization was conducted prior to tDCS to minimize the time between the end of tDCS stimulation and the performance of the main task (Fig. 1).

At the end of each session, participants completed a debriefing form (Reinhart et al., 2017) asking about their experience during the tDCS session (feelings of headache, difficulty in concentrating, changes in mood, changes in vision, fatigue, sensations of pain tingling, itching, or burning under electrode locations), and their judgment about whether they received active or sham stimulation at that session.

### Results

**Fig. 2:**
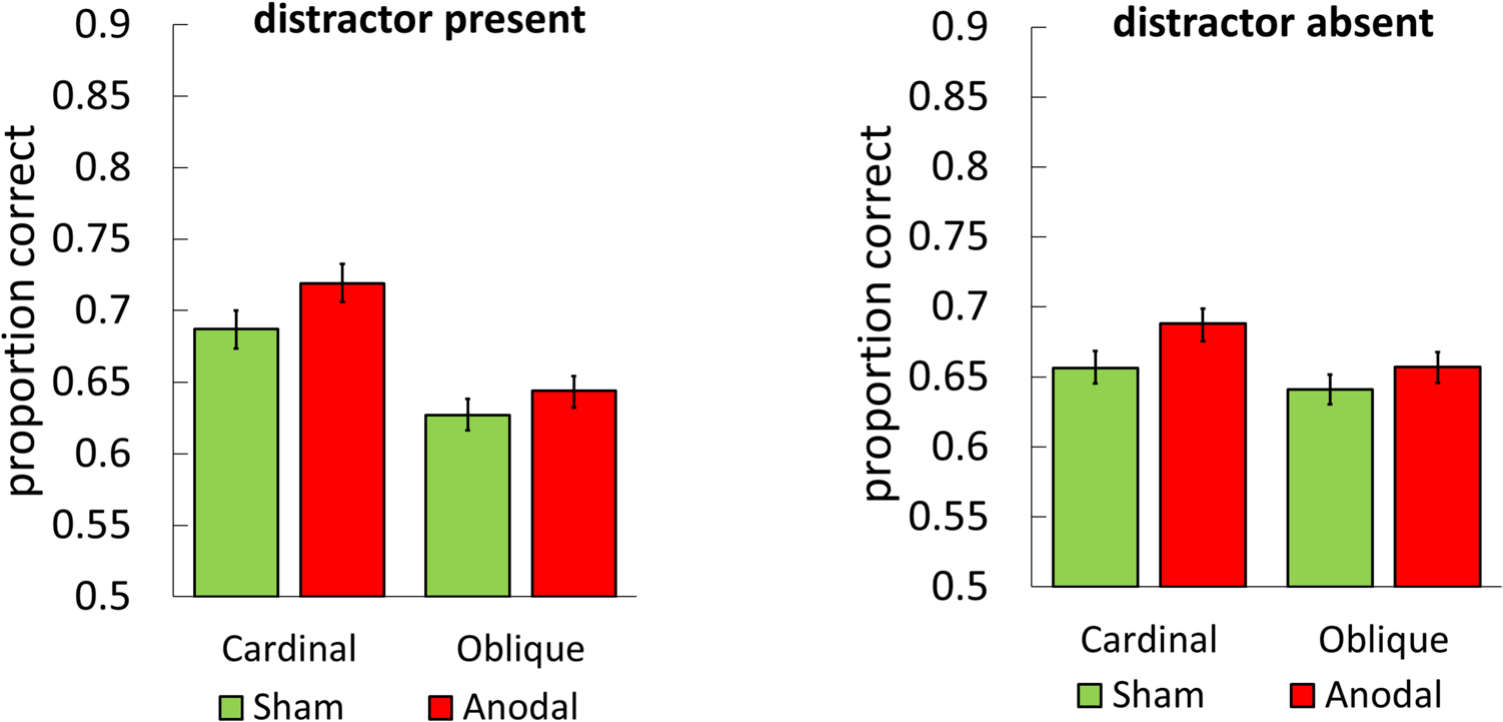
Comparison of VWM performance across anodal and sham tDCS sessions with the cardinal-oblique angle and distractor-presence conditions in Experiment 1

**Fig. 3:**
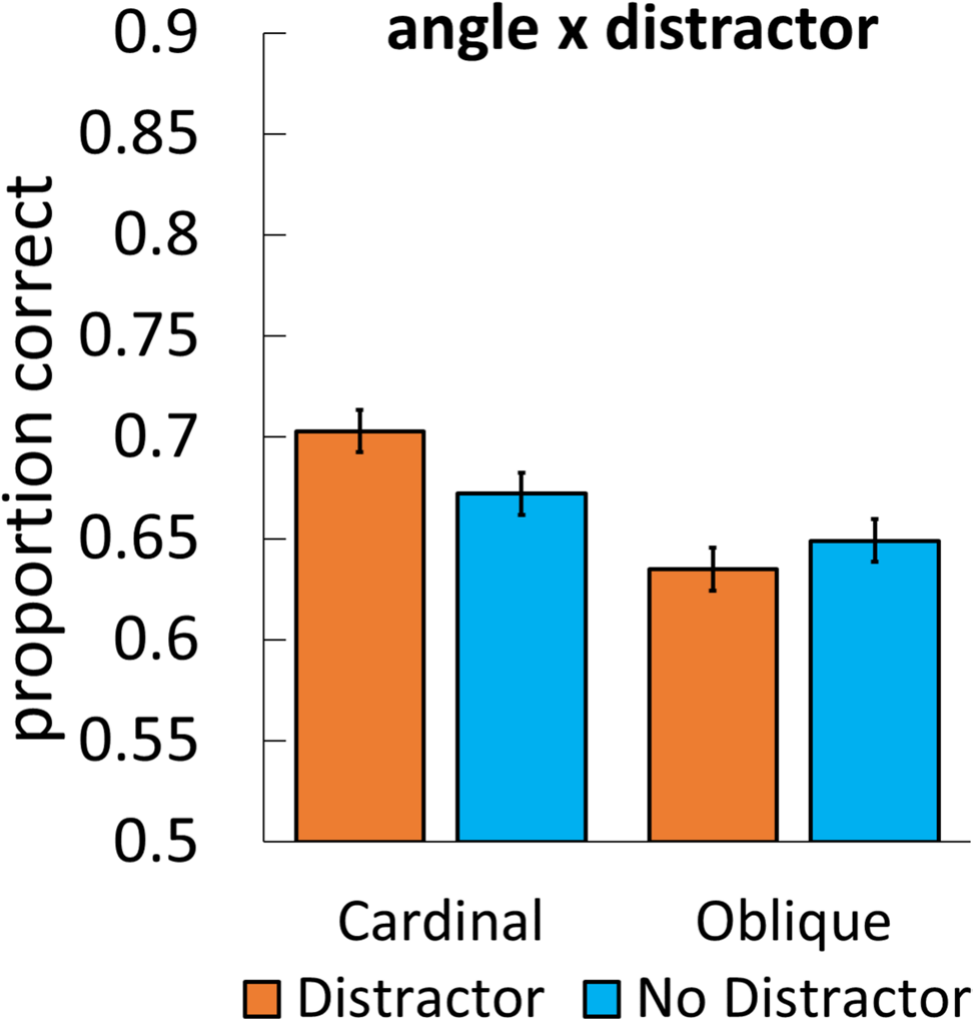
Interaction between oblique effect and distractor presence in Experiment 1 (pooled across anodal and sham tDCS conditions)

Performance was investigated by a three-way repeated-measures ANOVA with orientation angle (cardinal vs. oblique), distractor presence (present vs. absent), and tDCS condition (sham vs. anodal stimulation) based on the proportion of correct responses. Bayesian analyses specified coefficient priors for fixed and random terms (r scale fixed effects = 0.5, r scale random effects = 1), and a uniform model prior was chosen. We looked at the Bayes factors supporting inclusion of effects across matched models for Bayesian ANOVA, while model comparisons are presented in the Supplementary Materials Table S1. We primarily interpret the inclusion Bayes factors across matched models, because these indicate evidence for or against the relevance of a specific main effect or interaction without respect to other main effects or interactions in the model. There was an oblique effect—that is, an effect of target orientation, with cardinal angles being more accurate than oblique angles (BF_inc_ = 67.45). BFs suggested nearly 3:1 evidence against the effect of distractor-presence (BF_inc_ = 0.362), and there was neither convincing evidence for nor against a main effect of tDCS condition (BF_inc_ = 0.839), despite approximately matching the maximum planned sample size and exceeding the sample sizes of most of the past tDCS VWM experiments (Heimrath et al., 2012; Heinen et al., 2016; Hsu et al., 2016; Li et al., 2017; Robison et al., 2017; Tseng et al., 2012; Wang et al., 2019). Evidence for tDCS condition interactions with distractor presence (BF_inc_ = 0.525) or target orientation (BF_inc_ = 0.699) was insufficient to support clear conclusions based on Experiment 1 alone (but see *Pooled Analyses*, below). However, there was clear evidence against any three-way interaction between tDCS, distractor-presence, and target-orientation (BF_inc_ = 0.050) (Fig. 2; Table 1). Contrary to the hypothesized results, distractor-presence and target angle interacted strongly (BF_inc_ = 12.169), but surprisingly revealed a stronger oblique effect in distractor-present trials than distractor-absent trials (Fig. 3; Table 2).

**Table 1:** Experiment 1 analyses

	df	F	*p*	n^2^_p_	BF_inc_
Angle	1,34	14.933	<0.001	0.305	67.45
Distractor	1,34	1.65	0.208	0.046	0.362
tDCS	1,34	2.426	0.129	0.067	0.839
Distractor*angle	1,34	10.131	0.003	0.23	12.169
Distractor*tDCS	1,34	2.391x10^−4^	0.988	7.033x10^−6^	0.525
Angle*tDCS	1,34	1.525	0.225	0.043	0.699
tDCS*distractor*angle	1,34	0.002	0.966	5.461x10^−5^	0.050

**Table 2:** Simple main effects of angle (pooled across tDCS conditions)

	df	*t*	*p*	Cohen’s d	BF_inc_
Distractor-present	34	4.575	<0.001	0.773	395.468
Distractor-absent	34	1.894	0.067	0.32	0.897

Previous research suggested that anodal tDCS might only provide enhancement for the low-performing participants, but not the high-performers (Hsu et al., 2016). To test whether such individual differences explain the lack of tDCS effects, we conducted follow-up median-split analyses. The participants were split based on their overall performance pooled across all conditions. There was not a clear tDCS effect on the performance of the participants either above the overall median performance (BF_inc_ = 0.571) or below the overall median performance (BF_inc_ = 0.742) (Full median-split analyses are presented in Supplementary Materials, Tables S4 and S5), ruling out one possible explanation for the absence of tDCS effects in these data.

Based on the debriefing forms given at the end of each session, participants reported mild difficulty in concentrating; very mild to mild fatigue; and mild sensations of burning, tingling, or itching on the electrode locations. These reports were similar for anodal and sham sessions. Twenty-five of 34 (χ^2^ = 7.529, *p* = 0.006, BF_10_ = 9.358) participants believed that they received real stimulation in anodal sessions, and 18 of 34 (χ^2^ = 0.118, *p* = 0.732, BF_10_ = 0.223) participants believed that they received real stimulation in sham sessions (debriefing form results are missing for 1 participant) (tDCS side effect reports are presented in Supplementary Materials, Table S8). These results suggest that participants were not made very uncomfortable by tDCS, but they could successfully tell the difference between sham and stimulation; while blinding to tDCS condition is typically desirable, in this case, because tDCS effects on task performance were absent, detection of tDCS condition is somewhat reassuring that the lack of an effect cannot be explained by, e.g., equipment failure.

### Discussion

Experiment 1 showed no apparent enhancement of VWM by anodal tDCS on right PPC, and tDCS condition did not clearly interact with either distractor-presence or oblique effect, although we also lack strong evidence against any such interaction based on Experiment 1 alone. The strong oblique effect suggested that participants might still use the early visual cortex for VWM representations, because the early visual cortex is more sensitive to cardinal angles than the oblique angles (Berkley et al., 1975; Furmanski & Engel, 2000; Li et al., 2003). The stronger oblique effect under distraction might suggest that participants adopted a strategy to rely on the early visual cortex to protect VWM representations instead of PPC when they needed to ignore visual distractors.

## Experiment 2

In Experiment 2, we tested the effect of anodal tDCS on the occipital cortex. Especially during distraction with meaningful images, VWM reports appear to be correlated with IPS representations, while occipital representations were reliable in the absence of distraction by meaningful images (Bettencourt & Xu, 2016; Rademaker et al., 2019). We reasoned that tDCS to occipital cortex should thus affect VWM reports, in one of two ways: First, anodal tDCS could increase the sensitivity of occipital cortex to incoming visual inputs, making it more susceptible to visual distraction. Second, we predicted that, based on the sensory recruitment account of VWM, anodal tDCS stimulation should potentiate occipital cortex, enabling representation of a greater total amount of information in occipital cortex. Such increased representational capacity (summed across perception and memory) could improve VWM performance regardless of distraction, and could also limit the effects of distraction on VWM performance.

The preceding hypotheses also have implications for the oblique effect: With increased sensitivity to perceptual inputs, occipital cortex might be more selective to the cardinal angles and show a stronger oblique effect with anodal tDCS. Alternatively, with increased capacity, the occipital cortex could encode more fine-tuned and detailed representations for the oblique angles and show a decreased cardinal-oblique difference, that is, a decreased oblique effect.

Conversely, a frontoparietal account of VWM would predict that, with the increased sensitivity to distraction in the occipital cortex, VWM representations should be maintained in frontoparietal areas; therefore, we would not necessarily observe an oblique effect. This lack of oblique effect would not be explained by increased capacity for VWM and perception, rather it would be explained by the transfer of VWM representations from occipital cortex to the frontoparietal areas.

### Participants

Thirty-seven participants from the University of Houston community (3 males, 34 females, mean age 20.3) were recruited. The initial cohort consisted of 24 participants. After analysis of the main effect of tDCS, a Bayesian stopping rule was applied to reach Bayes factors that allow clear interpretations (BF > 5 for presence of effects or BF < 1/5 for absence of effects). Additional participant groups of 4 were recruited, and data collection was planned to be stopped when the sample size was increased by 50%. The main effect of tDCS was close to the suggested threshold for the absence of effect (BF = 0.231), but nevertheless, the maximal size stopping rule was applied after the planned maximum 50% increase in sample size. Four participants were excluded from the analyses for having chance-level performances in one of the experimental sessions (accuracy ≤ 50% overall); therefore, the following analyses were conducted with 33 participants (2 males, 31 females, mean age 20.21).

### Procedure

All task procedures were the same as Experiment 1, except that the anodal tDCS electrode was placed on the occipital cortex (Oz based on the 10/20 EEG system) on both active and sham stimulation conditions.

### Results

**Fig. 4:**
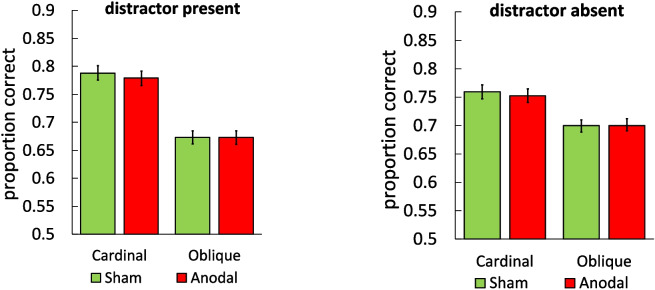
Comparison of VWM performance across anodal and sham tDCS sessions with the cardinal-oblique angle and distractor-presence conditions in Experiment 2

**Fig. 5:**
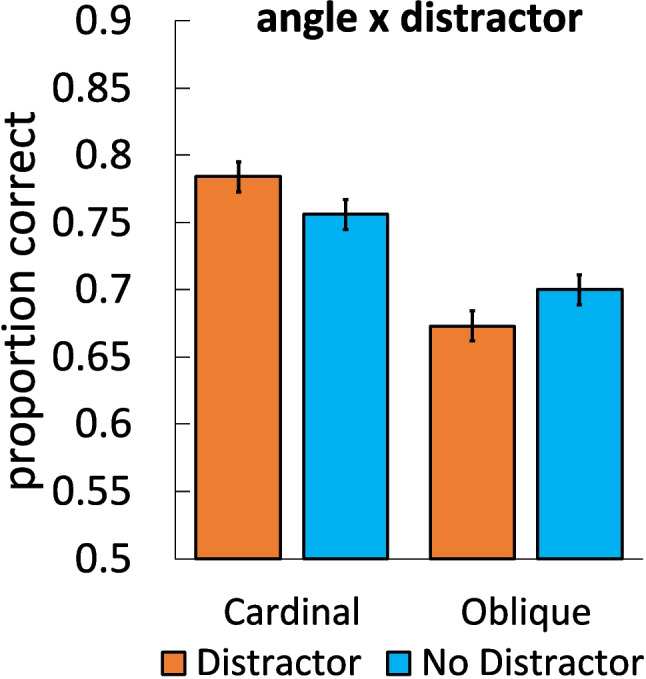
Interaction between oblique effect and distractor presence in Experiment 2 (pooled across anodal and sham tDCS conditions)

Performance was investigated by a three-way repeated-measures ANOVA with orientation angle (cardinal vs. oblique), distractor presence (present vs. absent), and tDCS condition (sham vs. anodal stimulation) based on the proportion of correct responses. As for Experiment 1, Bayesian analyses specified coefficient priors for fixed and random terms (r scale fixed effects = 0.5, r scale random effects = 1), a uniform model prior was chosen, we looked at the Bayes factor for inclusion of effects across matched models for Bayesian ANOVA, and model comparisons are presented in the supplementary materials (Table S2). There was a strong main effect of angle with cardinal angles being more accurate than oblique angles (BF_inc_ = 2.668x10^6^). Bayes factors provided approximately 3:1 evidence against the effect of distractor-presence (BF_inc_ = 0.347). There was no difference between performances across anodal and sham tDCS conditions (BF_inc_ = 0.231). It was not clear whether there was an interaction between tDCS conditions and oblique effect (BF_inc_ = 0.543). There was clearly no interaction between tDCS conditions and distractor presence (BF_inc_ = 0.072), nor was there a three-way interaction (BF_inc_ = 0.045) (Fig. 4; Table 3). The oblique effect was stronger with distractor presence, with increased accuracy for the cardinal angles compared with oblique angles under distractor presence (BF_inc_ = 6.406) (Fig. 5; Table 4).

**Table 3:** Experiment 2 analyses

	df	F	*p*	n^2^_p_	BF_inc_
Angle	1,33	63.112	<0.001	0.664	2.668x10^6^
Distractor	1,33	0.012	0.915	3.657x10^−4^	0.347
tDCS	1,33	0.066	0.798	0.002	0.231
tDCS*angle	1,33	0.276	0.603	0.009	0.543
tDCS*distractor	1,33	0.008	0.931	2.359x10^−4^	0.072
Distractor*angle	1,33	10.998	0.002	0.256	6.406
tDCS*distractor*angle	1,33	0.007	0.932	2.343x10^−4^	0.045

**Table 4:** Simple main effects of angle (pooled across tDCS conditions)

	df	*t*	*p*	Cohen’s d	BF_inc_
Distractor-present	32	7.313	<0.001	1.273	539969.084
Distractor-absent	32	4.916	<0.001	0.856	885.477

As for Experiment 1, follow-up median-split analyses were conducted. The participants were split based on their overall performance pooled across all conditions. Bayes factors did not provide clear tDCS effects on the performance of the participants either above the overall median performance (BF_inc_ = 0.555) or below the overall median performance (BF_inc_ = 0.378). (The full median-split analyses tables are presented in Supplementary Materials, Tables S6 and S7.)

Participants reported having mild difficulty in concentrating (BF = 1.084); and very mild to mild fatigue (BF = 0.199); and very mild to mild sensations of burning, tingling, or itching on the electrode locations (BF = 0.441), without clear differences in both anodal and sham sessions. Twenty-four of 33 (χ^2^ = 6.818, *p* = 0.009, BF_10_ = 6.551) participants believed that they received real stimulation in anodal sessions, and 20 of 33 participants (χ^2^ = 1.485, *p* = 0.223, BF_10_ = 0.441) believed that they received real stimulation in sham sessions (tDCS side effect reports are presented in Supplementary Materials, Table S9). As in Experiment 1, while we initially hoped that participants would be blind to tDCS condition, their reliable ability to detect the tDCS condition represents some reassurance that the absence of tDCS effects cannot be explained by, e.g., equipment failure.

### Discussion

Similar to Experiment 1, results showed no apparent enhancement with anodal tDCS on the occipital cortex, and tDCS condition did not interact with either distractor presence or oblique effect. As in Experiment 1, results of Experiment 2 revealed a strong interaction between oblique effect and distractor presence. Because of the similarity in the results of Experiments 1 and 2, we next pooled the experiments together to achieve greater sensitivity and more precise estimates of effect sizes in analyses of shared manipulations (i.e., manipulations of distraction and oblique vs. cardinal angles).

### Pooled analyses

#### Distractor presence and oblique effect interaction

**Fig. 6:**
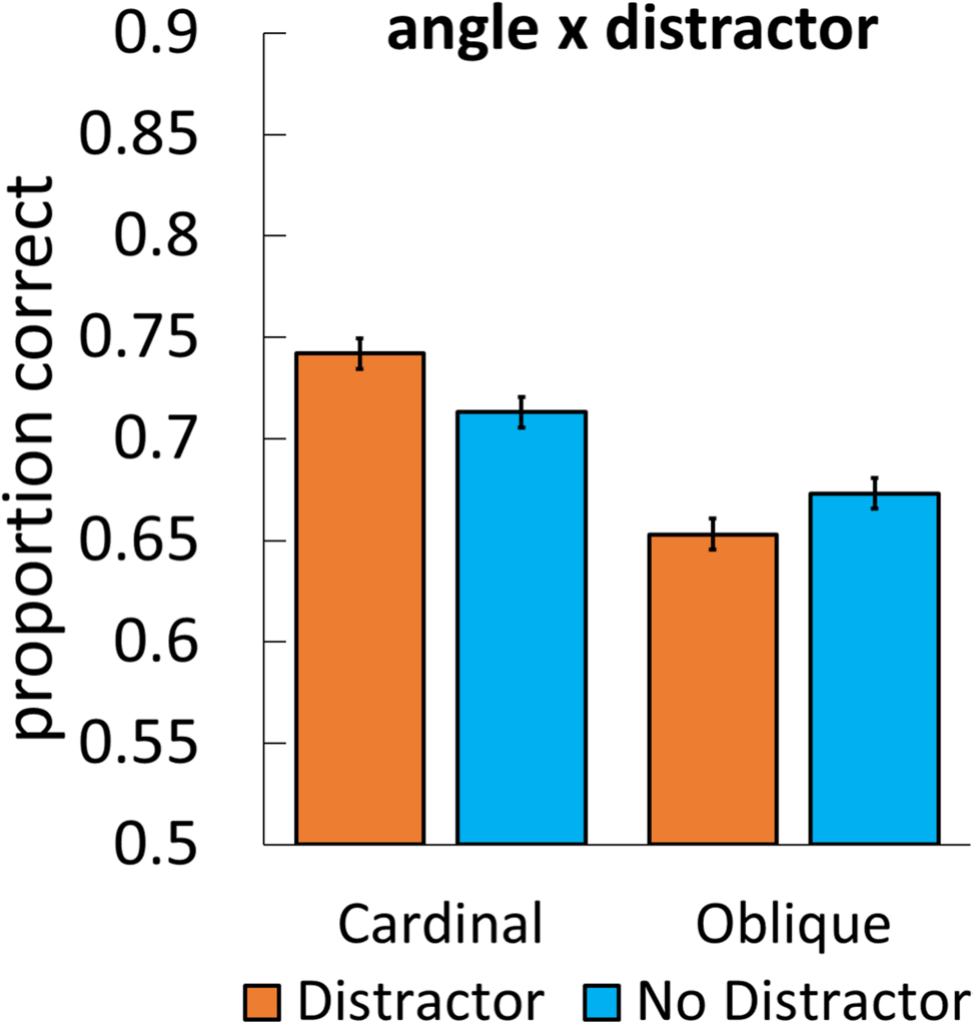
Interaction between oblique effect and distractor presence (pooled across Experiments 1 and 2, and anodal and sham tDCS conditions)

Both Experiments 1 and 2 revealed strong oblique effects, and the oblique effect was unexpectedly magnified by distraction. To our knowledge, the magnification of the oblique effect in working memory by distraction is a novel result. To more clearly understand this surprising interaction, a follow-up analyses was conducted with the data pooled across both experiments. Visual working memory performance was tested by a mixed ANOVA with orientation angle (cardinal vs. oblique), distractor presence (present vs. absent), and tDCS condition (sham vs. anodal stimulation) as within-subjects factors, and experiment (parietal tDCS in Experiment 1 vs. occipital tDCS in Experiment 2) as a between-subjects factor. As for the individual experiment analyses, we looked at the Bayes factors for inclusion of effects across matched models for Bayesian ANOVA, and model comparisons are presented in the Supplementary Materials (Table S3). As expected, there was weak evidence against the difference between anodal (pooled across parietal and occipital targets) and sham tDCS conditions (BF_inc_ = 0.547). There was approximately 5:1 evidence against the main effect of distractor presence (BF_inc_ = 0.197), and there was very strong evidence for the main effect of angle with higher accuracy for cardinal than oblique orientations (BF_inc_ = 1.968x10^8^). The lack of interactions between tDCS condition and distractor presence (BF_inc_ = 0.159) and tDCS condition and oblique effect (BF_inc_ = 0.195) provided evidence that tDCS did not modulate either effect. There was a strong interaction between distractor-presence and oblique effect (BF_inc_ = 1021.671), with larger oblique effect for the distractor-present conditions than the distractor-absent conditions (Fig. 6; Tables 5 and 6). Overall, these results strengthen the evidence for an increased oblique effect in VWM under visual distraction.

**Table 5:** Mixed ANOVA across experiments 1 and 2

	df	F	*p*	n^2^_p_	BF_inc_
Experiment	1,66	4.894	0.03	0.069	1.698
Angle	1,66	66.03	<0.001	0.5	1.968x10^8^
Angle*Exp	1,66	5.612	0.021	0.078	2.87
Distractor	1,66	1.020	0.316	0.015	0.197
Distractor*Exp	1,66	0.744	0.391	0.011	0.233
tDCS	1,66	0.819	0.369	0.012	0.547
tDCS*Exp	1,66	1.622	0.207	0.024	0.553
tDCS*angle	1,66	0.117	0.733	0.002	0.195
tDCS*angle*Exp	1,66	1.389	0.243	0.021	0.253
tDCS*distractor	1,66	0.006	0.941	8.463x10^−5^	0.159
tDCS*distractor*Exp	1,66	0.003	0.957	4.394x10^−5^	0.273
Distractor*angle	1,66	21.215	<0.001	0.243	1021.671
Distractor*angle*Exp	1,66	0.268	0.606	0.004	0.308
tDCS*distractor*angle	1,66	0.008	0.928	1.238x10^−4^	0.217
tDCS*dist*angle*Exp	1,66	7.651x10^−4^	0.978	1.159x10^−5^	0.39

**Table 6:** Simple main effects of angle (pooled across tDCS conditions)

	df	*t*	*p*	Cohen’s d	BF_inc_
Distractor-present	67	8.181	<0.001	0.992	7.931x10^8^
Distractor-absent	67	4.546	<0.001	0.551	800.231

## General discussion

The present study largely contained null results with respect to tDCS, yet revealed multiple positive findings with respect to behavior. We first discuss the null tDCS findings and interpret them in light of past weak evidence for the effectiveness of tDCS to enhance VWM, as well as discuss possible accounts of representation in VWM in light of our results. We then discuss the oblique effect’s interactions with distraction.

### Ineffectiveness of tDCS to enhance VWM precision

The present research aimed to investigate potential causal relationships between parietal or occipital representations and VWM performance and furthermore how the enhancement of neural resources underlying VWM might interact with the stimulus features and task demands. Contrary to our hypotheses, neither occipital nor right PPC anodal tDCS enhanced VWM performance, either overall or in narrower task-specific conditions. Thus, this research adds to the list of studies questioning the effectiveness of PPC (and also occipital) tDCS to enhance VWM. Notably, previous tDCS studies aiming to enhance VWM performance usually tested VWM capacity in terms of number of the objects stored (Heimrath et al., 2012; Heinen et al., 2016; Hsu et al., 2016; Tseng et al., 2012). However, there have been mixed findings (Dumont et al., 2021; Jiang et al., 2024; Robison et al., 2017). The present study thus broadens this past research to demonstrate the ineffectiveness of tDCS to enhance VWM measured by representational precision (ability to make fine-grained orientation discriminations at small set size), not just storage capacity.

Whether tDCS can effectively manipulate VWM performance might be contingent on the task design and sensitivity of the cognitive measures (Reinhart et al., 2017). Especially, effects of tDCS might depend on whether the task demands the participants go over their cognitive load capacity (Roe et al., 2016). However, it is unlikely that manipulations of task conditions and demands are the reason for ineffectiveness of tDCS in this study. Even though the item load was not high in the study, we required difficult fine-grained judgements of orientation from memory. That we found a strong oblique effect, and that there was a robust modulation of the oblique effect by the presence of distractors, suggests that our task was sensitive enough to reveal gradations of memory performance.

Other studies have suggested that effectiveness of tDCS might be bound to individual differences, with low performers benefiting the most from tDCS (Hsu et al., 2016; Tseng et al., 2012; Vergallito et al., 2022). Even though individual differences may explain tDCS effects (or their absence) in some circumstances, analyses comparing low vs. high performers on the present task indicate that individual differences do not explain the ineffectiveness of tDCS.

We also are confident that the ineffectiveness of tDCS was not simply due to insensitivity of our study design: each of Experiments 1 and 2 had larger sample sizes compared with prior tDCS studies in the VWM literature (ranging from 10 to 24 participants per experiment; Heimrath et al., 2012; Heinen et al., 2016; Hsu et al., 2016; Li et al., 2017; Robison et al., 2017; Tseng et al., 2012; Wang et al., 2019), and we also gained sensitivity by using a within-subjects design in which each participant received both anodal and sham sessions, contrary to some previous studies but consistent with best-practices recommendations (Reinhart et al., 2017). During the task design and application of tDCS, we carefully followed the available guidelines and used appropriate and standard tDCS parameters (2-mA intensity, 20-min stimulation duration, and offline testing of behavior) (Reinhart et al., 2017; Thair et al., 2017).

One consideration is that tDCS is only coarsely targeted to specific brain regions, and it lacks spatial specificity. Individualized localization of the brain regions with structural MRI was beyond the means of our research facilities; therefore, we opted for using 10/20 EEG system to target posterior parietal cortex and occipital cortex. However, it is unlikely that the tDCS procedure failed to stimulate the targeted brain locations due to the widespread effects of tDCS stimulation. Moreover, the connection quality of the tDCS device was monitored at the beginning, at the middle, and at the end of every tDCS session. Whether the participants felt the tickling sensation was verbally confirmed every task session and also via debriefing forms at the end of each session (see Supplementary Materials Tables S5-S6). Even though participants reported similar sensations of tickling across anodal and sham sessions, reports in the debriefing forms suggested that participants could tell that they received real stimulation in anodal sessions, although they were uncertain whether it was real or placebo stimulation in the sham sessions. Together, these suggest that the null tDCS results do not simply stem from ineffective application of tDCS. In addition, this study aimed to test role of occipital cortex on VWM representations as in the flexible form of sensory recruitment model, instead of a specific subregion of the visual cortex (Ester et al., 2009; Rademaker et al., 2019). Similarly, we tested the role of parietal areas to investigate the role of frontoparietal account of VWM (Ester et al., 2015; Roth et al., 2006) and not a specific sub-region in the parietal cortex.

One alternative to test the causal effect of neural modulation on VWM performance could be using transcranial alternating current stimulation (tACS) instead of tDCS. tDCS deploys either anodal or cathodal stimulation unidirectionally. Conversely, tACS alternates between anodal and cathodal poles at a specified frequency in bidirectional manner and modulates frequency-specific cortical activity and brain oscillations (Antal & Herrmann, 2016; Grover et al., 2023). For example, right PPC can be targeted at a 4Hz slow (theta) frequency, which is associated with VWM capacity, to modulate VWM performance (Bender et al., 2019). However, the mechanisms by which tACS acts are not completely understood and the literature on the effects of tACS on visual cognition is also in its infancy (Antal & Herrmann, 2016; Grover et al., 2023). Moreover, it is not entirely clear whether tACS should be expected to lead to consistent enhancement or detrimental effects unless its phase is properly aligned with stimulus-entrained or endogenous oscillatory activity in each participant, potentially making effective use of tACS a more challenging endeavor and motivating the use of simpler techniques, such as tDCS. Nevertheless, future studies should apply this technique using similar task designs as the current study.

There are two potential limitations of the tDCS technology that might have caused the lack of tDCS effect in this study. First, participant factors beyond the control of the experimenter, such as hair thickness, amount of sweat produced on the target location, and skull thickness, might affect the effectiveness of tDCS (Horvath et al., 2014; Thair et al., 2017). The majority of the participant sample in Experiments 1 and 2 consisted of female participants, many of whom appeared to have thick and dense hair. One solution proposed for this issue is place the electrodes on scalp with electro-conductive gel instead of saline soaked sponges (Thair et al., 2017); however, that method is not feasible, because it causes higher discomfort to the participants. Regardless, as noted, this is unlikely to explain our results, because connection strength was monitored throughout the manipulation. Second, tDCS might have limited effects to improve cognitive performance in a young and healthy participant population (Dumont et al., 2021; Thair et al., 2017). Our participants had a mean age of approximately 20, and all the participants were screened for neurological disorders and psychiatric diagnoses via self-report. Future studies should explore whether tDCS may be effective in older populations or populations of disorder.

Finally, one design choice for the delayed orientation report task in Experiments 1 and 2 might have potentially affected the results: Distractor-present and distractor-absent conditions were presented in separate blocks of trials, instead of distractor-present and -absent trials being randomly intermixed throughout the experiments. The goal of blocking the distractor-present and -absent trials was to make distractors predictable (Bettencourt & Xu, 2016; Xu, 2020) and thus to test whether the participants would strategically utilize the neural resources (parietal or occipital) under investigation. However, the blocked nature of the study might have led the participants to spend overall less effort to store more detailed VWM representation and biased their responses towards cardinal and away from oblique angles, by encoding more categorical representations (e.g. verbal labels for vertical and horizontal orientations), rather than fine-tuned visual representations.

#### Neural resources under distraction

One goal of this study was to investigate how the early visual cortex can store VWM representations during ongoing perceptual distraction. Previous studies testing this claim found that task-irrelevant visual distractors interfered with the neural code of the VWM representations in the early visual cortex, but not in parietal cortex (Bettencourt & Xu, 2016; Xu, 2017), even though behavior was not (Bettencourt & Xu, 2016) or was minimally (Rademaker et al., 2019) disrupted by distraction. One possibility is, disruption of the neural code does not mean complete loss of representation; instead it might change the format of the representation and cause systematic biases in VWM (Hallenbeck et al., 2021). Indeed, the interaction between the oblique effect and distractor presence might be interpreted as a change in representational format and a systematic bias to cardinal orientations (Bae, 2021, 2024), but not as a total disruption. This leaves open the question, how did distraction change the representation? Could it have led to changes in the reliance on an occipital sensory code with overrepresentation of cardinal angles versus an abstract parietal code that may be more categorical?

Hallenbeck & colleagues (2021) found that the neural code that is found in both sensory and parietal cortices gets disrupted under distraction during memory delay, but it is not a total loss, and those neural codes can be recovered at the end of VWM delay across both regions. Therefore, VWM content can be protected against distraction with the distributed resources of VWM and not only by frontoparietal areas (Hallenbeck et al., 2021). They also reported that distraction caused a specific trial-to-trial bias systematically, with memory errors being correlated with representations in the early visual cortex rather than association cortex. Therefore, the increased oblique effect in the present study might reflect a recovery of the sensory code after distraction, with a sensory code in the visual cortex that is more sensitive to the cardinal orientations. This possibility is also supported by the idea that a sensory code is specifically important for target-probe comparison, even when it is not essential for storage of VWM content in all cases (Xu, 2020). This idea is also consistent with evidence showing recovery of neural signatures of VWM when attention is refocused on the contents of short term memory, even after the neural signal is lost for the unattended information (Lewis-Peacock et al., 2012).

Another possibility is that, during distraction, VWM representations might be retained in an abstract format via a parietal code. Abstract VWM information is represented in frontoparietal regions with less sensory detail and such representations carry more categorical information or may even entail verbal codes (e.g., “horizontal” and “vertical” verbal labels for cardinal orientations) (Christophel et al., 2017). Therefore, it is also possible that the task-irrelevant visual distractors in the present study interfered with the sensory code and caused frontoparietal regions to protect VWM content (Lorenc et al., 2021). If the distractors interfered with the sensory code, and the parietal code is the basis for a recovery after distraction, participants’ reliance on the abstract memory format and accompanying loss of the details of oblique orientations could result in biasing towards cardinal orientations. It should be noted that, at least at first glance, this account appears to contradict past research (Berkley et al., 1975; Furmanski & Engel, 2000; Li et al., 2003), which attributed the oblique effect to occipital cortex in low-level visual perception. It is possible that sensory oblique effect and memory oblique effect might have different neural bases. Nevertheless, further studies are needed to investigate whether the sensory or parietal code is the basis for responding after distraction, with a task design that directly tests the two possibilities and with direct neural correlate measures. The present approach, which used tDCS to modulate occipital and parietal cortex, did not reveal such results. An intriguing possibility is that the very flexibility of VWM to guide behavior based on a range of representations in different brain regions led to our null tDCS results and our subtle (interaction with oblique effect) distraction results. Perhaps there is little benefit or cost to modulation of just one brain region supporting VWM, which is consistent with parallel coding and the distributed networks account of VWM representation, and would suggest that future studies should jointly apply simultaneous stimulation to occipital and parietal cortex. This idea of concurrent stimulation of multiple cortical areas was similarly proposed in other brain stimulation methods, such as TMS (Phylactou et al., 2024) and ultrasound (Jones et al., 2022), and has been implemented using high definition tDCS (Huang et al., 2025).

#### Oblique effect in VWM

Our results demonstrated a robust oblique effect in VWM, with the cardinal angles remembered more accurately than the oblique angles. Oblique effects for VWM representations were previously observed in the literature. Specifically, an oblique effect in VWM was consistently observed regardless of the VWM load, across set sizes of 1, 2, 3, and 6 (Pratte et al., 2017). Similar oblique effects for VWM tasks have been reported in EEG studies, regardless of the classification accuracy of cardinal and oblique angles (Bae, 2021). Surprisingly, we observed that the oblique effect was enhanced by the presence of task-irrelevant sensory distractors, which, to our knowledge, is a novel result. The oblique effect is thought to be based on the better tuning for the cardinal orientations than the oblique orientations in early visual cortex (Berkley et al., 1975; Furmanski & Engel, 2000; Li et al., 2003). Keogh & colleagues argued that the lack of oblique effect observed in the VWM representations of their aphantasic participants stemmed from the use of task strategies that rely on parietal cortex (Keogh et al., 2021). Considering this reasoning, we suggest that the strong oblique effect observed in the present study could reflect the use of sensory codes in the early visual cortex for VWM representations, adding more evidence for the sensory recruitment account. While we presented distractor-present and distractor-absent trials in separate blocks, it is unlikely that participants adopted very different strategies during distractor-present blocks, as there was evidence against a main effect of distractor presence.

## Conclusions

Our goal with this study was to investigate the sensory recruitment, frontoparietal, and distributed networks accounts of VWM, and whether, depending on stimuli and task conditions, those resources can be used flexibly. We sought to find direct causal evidence using neural modulation for the roles of different brain regions in VWM performance under varying stimuli properties and task demands. Even though anodal tDCS on parietal and occipital cortices was ineffective, the results revealed a strong oblique effect in VWM and modulation of the cardinal orientation bias by task-irrelevant visual distractors. Overall, the absence of tDCS effects is most consistent with parallel coding in distributed networks that is resistant to exogenous manipulation of any one cortical region, while current evidence from the oblique effect could suggest reliance on the sensory code in the early visual cortex to maintain VWM representations even under distraction, which is most consistent with the sensory recruitment account.

## Supplementary Information

Below is the link to the electronic supplementary material.

Supplementary file1 (DOCX 35 KB)

## Data Availability

Data and materials will be made available on OSF (https://osf.io/5cnjt/) upon acceptance of the manuscript.

## Footnotes

1 The rotation was 8 degrees for the first eight participants; however, it was increased to 12 degrees to avoid floor performance.
