# Supplementary material for: The oblique effect in visual working memory is enhanced by distraction, regardless of tDCS manipulations
Source: Cogn Affect Behav Neurosci. 2026 Apr 21;26(4):1597–612. doi: 10.3758/s13415-026-01443-z (PMC13385089; doi:10.3758/s13415-026-01443-z)
Supplement: Supplementary file 1 — Supplementary file1 (DOCX 35 KB) [file 13415_2026_1443_MOESM1_ESM.docx]

| **BAYESIAN MODEL COMPARISONS**  Table S1. Experiment 1 Model Comparison | | | | | | | | | | | | | | |
| --- | --- | --- | --- | --- | --- | --- | --- | --- | --- | --- | --- | --- | --- | --- |
| Models | | P(M) | | | P(M\|data) | | BF_M_ | | | BF_10_ | | error % | | |
| Distractor + Angle + Distractor ✻  Angle |  | | 0.053 |  | | 0.275 |  | 6.837 |  | 1.000 |  |  |  |  |
| tDCS + Distractor + Angle + Distractor ✻  Angle |  | | 0.053 |  | | 0.231 |  | 5.392 |  | 0.837 |  | 6.956 |  |  |
| tDCS + Distractor + Angle + tDCS ✻  Distractor + tDCS ✻  Angle + Distractor ✻  Angle |  | | 0.053 |  | | 0.118 |  | 2.414 |  | 0.430 |  | 81.152 |  |  |
| tDCS + Distractor + Angle + tDCS ✻  Angle + Distractor ✻  Angle |  | | 0.053 |  | | 0.090 |  | 1.788 |  | 0.328 |  | 7.511 |  |  |
| Angle |  | | 0.053 |  | | 0.062 |  | 1.195 |  | 0.226 |  | 6.278 |  |  |
| tDCS + Distractor + Angle + tDCS ✻  Distractor + Distractor ✻  Angle |  | | 0.053 |  | | 0.058 |  | 1.113 |  | 0.212 |  | 8.671 |  |  |
| tDCS + Angle |  | | 0.053 |  | | 0.055 |  | 1.055 |  | 0.201 |  | 7.500 |  |  |
| tDCS + Angle + tDCS ✻  Angle |  | | 0.053 |  | | 0.038 |  | 0.706 |  | 0.137 |  | 38.515 |  |  |
| Distractor + Angle |  | | 0.053 |  | | 0.027 |  | 0.508 |  | 0.100 |  | 22.415 |  |  |
| tDCS + Distractor + Angle |  | | 0.053 |  | | 0.020 |  | 0.375 |  | 0.074 |  | 9.024 |  |  |
|  | | | | | | | | | | | | | | |
| Note.  All models include subject, and random slopes for all repeated measures factors. Showing the best 10 out of 19 models. | | | | | | | | | | | | | | |
| Table S2. Experiment 2 Model Comparison | | | | | | | | | | | | | |  |
| Models | | | P(M) | | | P(M\|data) | BF_M_ | | | BF_10_ | | error % | |  |
| Distractor + Angle + Distractor ✻  Angle |  | | 0.053 |  | | 0.668 |  | 36.212 |  | 1.000 |  |  |  |  |
| Angle |  | | 0.053 |  | | 0.165 |  | 3.568 |  | 0.248 |  | 8.057 |  |  |
| tDCS + Distractor + Angle + Distractor ✻  Angle |  | | 0.053 |  | | 0.089 |  | 1.751 |  | 0.133 |  | 58.944 |  |  |
| Distractor + Angle |  | | 0.053 |  | | 0.031 |  | 0.579 |  | 0.047 |  | 8.245 |  |  |
| tDCS + Distractor + Angle + tDCS ✻  Distractor + Distractor ✻  Angle |  | | 0.053 |  | | 0.022 |  | 0.401 |  | 0.033 |  | 71.394 |  |  |
| tDCS + Angle + tDCS ✻  Angle |  | | 0.053 |  | | 0.011 |  | 0.198 |  | 0.016 |  | 79.376 |  |  |
| tDCS + Distractor + Angle + tDCS ✻  Angle + Distractor ✻  Angle |  | | 0.053 |  | | 0.007 |  | 0.126 |  | 0.010 |  | 52.086 |  |  |
| tDCS + Angle |  | | 0.053 |  | | 0.003 |  | 0.060 |  | 0.005 |  | 58.749 |  |  |
| tDCS + Distractor + Angle |  | | 0.053 |  | | 0.002 |  | 0.036 |  | 0.003 |  | 82.130 |  |  |
| tDCS + Distractor + Angle + tDCS ✻  Angle |  | | 0.053 |  | | 9.440×10^-4^ |  | 0.017 |  | 0.001 |  | 42.362 |  |  |
|  | | | | | | | | | | | | | |  |
| Note.  All models include subject, and random slopes for all repeated measures factors. Showing the best 10 out of 19 models. | | | | | | | | | | | | | |  |
|  | | | | | | | | | | | | | |  |

| *Table S3. Model Comparison for pooled analysis* | | | | | | | | | | | |
| --- | --- | --- | --- | --- | --- | --- | --- | --- | --- | --- | --- |
| Models | | P(M) | | P(M\|data) | | BF_M_ | | BF_10_ | | error % | |
| Distractor + Angle + Exp + Distractor ✻  Angle + Angle ✻  Exp |  | 0.006 |  | 0.239 |  | 52.099 |  | 1.000 |  |  |  |
| tDCS + Distractor + Angle + Exp + Distractor ✻  Angle + Angle ✻  Exp |  | 0.006 |  | 0.099 |  | 18.142 |  | 0.412 |  | 16.232 |  |
| Distractor + Angle + Exp + Distractor ✻  Angle |  | 0.006 |  | 0.087 |  | 15.888 |  | 0.366 |  | 13.688 |  |
| Distractor + Angle + Exp + Distractor ✻  Angle + Distractor ✻  Exp + Angle ✻  Exp |  | 0.006 |  | 0.073 |  | 13.119 |  | 0.307 |  | 19.634 |  |
| tDCS + Distractor + Angle + Exp + Distractor ✻  Angle + tDCS ✻  Exp + Angle ✻  Exp |  | 0.006 |  | 0.064 |  | 11.410 |  | 0.269 |  | 30.430 |  |
| Distractor + Angle + Distractor ✻  Angle |  | 0.006 |  | 0.063 |  | 11.199 |  | 0.265 |  | 14.692 |  |
| tDCS + Distractor + Angle + Exp + Distractor ✻  Angle + tDCS ✻  Exp |  | 0.006 |  | 0.051 |  | 8.886 |  | 0.213 |  | 58.339 |  |
| tDCS + Distractor + Angle + Exp + Distractor ✻  Angle |  | 0.006 |  | 0.040 |  | 6.832 |  | 0.165 |  | 15.701 |  |
| Distractor + Angle + Exp + Distractor ✻  Angle + Distractor ✻  Exp |  | 0.006 |  | 0.033 |  | 5.658 |  | 0.138 |  | 22.303 |  |
| tDCS + Distractor + Angle + Exp + Distractor ✻  Angle + Distractor ✻  Exp + Angle ✻  Exp |  | 0.006 |  | 0.023 |  | 3.902 |  | 0.096 |  | 15.933 |  |
|  | | | | | | | | | | | |
| *Note.*  All models include subject, and random slopes for all repeated measures factors. | | | | | | | | | | | |
| *Note.*  Showing the best 10 out of 167 models. | | | | | | | | | | | |

**MEDIAN-SPLIT ANALYSES FOR EXPERIMENTS 1 & 2**

Table S4. Experiment 1 Median-split with high-performers

|  | df | F | *p* | n^2^_p_ | BF_inc_ |
| --- | --- | --- | --- | --- | --- |
| Angle | 1,17 | 17.770 | <0.001 | 0.511 | 61.410 |
| Distractor | 1,17 | 7.785 | 0.013 | 0.314 | 2.025 |
| tDCS | 1,17 | 0.975 | 0.337 | 0.054 | 0.571 |
| Distractor*angle | 1,17 | 42.751 | <0.001 | 0.715 | 864.920 |
| Distractor*tDCS | 1,17 | 0.710 | 0.411 | 0.040 | 0.361 |
| Angle*tDCS | 1,17 | 0.004 | 0.948 | 2.583x10^-4^ | 0.388 |
| tDCS*distractor*angle | 1,17 | 0.145 | 0.708 | 0.008 | 0.347 |

Table S5. Experiment 1 Median-split with low-performers

|  | df | F | *p* | n^2^_p_ | BF_inc_ |
| --- | --- | --- | --- | --- | --- |
| Angle | 1,16 | 1.668 | 0.215 | 0.094 | 0.656 |
| Distractor | 1,16 | 0.194 | 0.666 | 0.012 | 0.304 |
| tDCS | 1,16 | 1.404 | 0.253 | 0.081 | 0.742 |
| Distractor*angle | 1,16 | 0.039 | 0.847 | 0.002 | 0.405 |
| Distractor*tDCS | 1,16 | 0.405 | 0.533 | 0.025 | 0.396 |
| Angle*tDCS | 1,16 | 4.780 | 0.044 | 0.230 | 1.025 |
| tDCS*distractor*angle | 1,16 | 0.249 | 0.625 | 0.015 | 0.372 |

Table S6. Experiment 2 Median-split with high-performers

|  | df | F | *p* | n^2^_p_ | BF_inc_ |
| --- | --- | --- | --- | --- | --- |
| Angle | 1,18 | 60.478 | <0.001 | 0.771 | 73109 |
| Distractor | 1,18 | 0.218 | 0.646 | 0.012 | 0.257 |
| tDCS | 1,18 | 0.003 | 0.959 | 1.512x10^-4^ | 0.555 |
| Distractor*angle | 1,18 | 8.779 | 0.008 | 0.328 | 5.784 |
| Distractor*tDCS | 1,18 | 1.037 | 0.322 | 0.054 | 0.448 |
| Angle*tDCS | 1,18 | 1.270 | 0.275 | 0.066 | 0.513 |
| tDCS*distractor*angle | 1,18 | 0.432 | 0.519 | 0.023 | 0.736 |

Table S7. Experiment 2 Median-split with low-performers

|  | df | F | *p* | n^2^_p_ | BF_inc_ |
| --- | --- | --- | --- | --- | --- |
| Angle | 1,13 | 14.651 | 0.002 | 0.530 | 22.370 |
| Distractor | 1,13 | 0.183 | 0.676 | 0.014 | 0.435 |
| tDCS | 1,13 | 0.092 | 0.766 | 0.007 | 0.378 |
| Distractor*angle | 1,13 | 3.004 | 0.107 | 0.188 | 0.847 |
| Distractor*tDCS | 1,13 | 1.192 | 0.295 | 0.084 | 0.289 |
| Angle*tDCS | 1,13 | 0.185 | 0.674 | 0.014 | 0.513 |
| tDCS*distractor*angle | 1,13 | 0.312 | 0.586 | 0.023 | 3.589 |

**TDCS SIDE EFFECT REPORTS**

Table S8. tDCS side effects Experiment 1

| Session | Headache | Difficulty Concentrating | Change in Mood | Change in vision | Fatigue | Sensation (burning, tingling, itching |
| --- | --- | --- | --- | --- | --- | --- |
| Anodal | 0.529 (0.165) | 2.412  (0.212) | 0.74 (0.183) | 0.618 (0.184) | 1.235 (0.260) | 2.059 (0.184) |
| Sham | 0.324 (0.125) | 1.882  (0.273) | 0.500 (0.165) | 0.294 (0.108) | 1.294 (0.252) | 1.618 (0.174) |
| t | 1.190 | 1.769 | 1.407 | 1.874 | -0.250 | 2.218 |
| *p* | 0.242 | 0.086 | 0.169 | 0.070 | 0.804 | 0.034 |
| BF | 0.352 | 0.745 | 0.451 | 0.876 | 0.189 | 1.574 |

Upper part of the table shows the mean (SE) statistics for each question on the debriefing form. Lower part of the table compares the responses across anodal and sham sessions. Response scales are ranging from 0 to 5. 0 = None, 1 = Very Mild, 2 = Mild, 3 = Moderate, 4 = Severe, 5 = Extreme

Table S9. tDCS side effects Experiment 2

| Session | Headache | Difficulty Concentrating | Change in Mood | Change in vision | Fatigue | Sensation (burning, tingling, itching |
| --- | --- | --- | --- | --- | --- | --- |
| Anodal | 0.485 (0.138) | 2.091 (0.240) | 0.455 (0.151) | 0.424 (0.185) | 1.697 (0.244) | 1.879 (0.183) |
| Sham | 0.333 (0.112) | 1.758 (0.226) | 0.545 (0.180) | 0.273 (0.139) | 1.606 (0.246) | 1.576 (0.180) |
| t | 0.961 | 2.000 | -0.399 | 0.961 | 0.373 | 1.379 |
| *p* | 0.344 | 0.54 | 0.692 | 0.344 | 0.712 | 0.177 |
| BF | 0.285 | 1.084 | 0.201 | 0.285 | 0.199 | 0.441 |

Upper part of the table shows the mean (SE) statistics for each question on the debriefing form. Lower part of the table compares the responses across anodal and sham sessions. Response scales are ranging from 0 to 5. 0 = None, 1 = Very Mild, 2 = Mild, 3 = Moderate, 4 = Severe, 5 = Extreme
